# Investigation of the efficacy and pharmacological mechanism of Danhong injections for treating chronic obstructive pulmonary disease: A PRISMA-compliant meta-analysis and network pharmacology analysis

**DOI:** 10.1097/MD.0000000000032846

**Published:** 2023-02-03

**Authors:** Xiaoyu Gao, Jinsong Gao

**Affiliations:** a Department of Pharmacy, Jiangnan Hospital Affiliated to Zhejiang University of Traditional Chinese Medicine Xiaoshan Hospital of Traditional Chinese Medicine, Hangzhou, Zhejiang, China; b Intensive Care Unit, Jiangnan Hospital Affiliated to Zhejiang University of Traditional Chinese Medicine Xiaoshan Hospital of Traditional Chinese Medicine, Hangzhou, Zhejiang, China.

**Keywords:** chronic obstructive pulmonary disease, Danhong injection, meta-analysis, network pharmacology

## Abstract

Accumulating evidence supported the clinical efficacy of Danhong injection (DHI) on chronic obstructive pulmonary disease (COPD). It is urgent to summarize the effects of DHI on various outcomes in COPD patients and to elucidate the molecular mechanisms of DHI in treating COPD.

**Methods:**

Eligible studies were retrieved from 6 databases including China national knowledge infrastructure, Wangfang, VIP, web of science, PubMed, and Embase. The heterogeneity across studies was tested using the *I*^2^ statistic and the quality of studies was assessed. The pooled evaluation of outcomes was calculated using a fix- or random-effect model according to the heterogeneity. The underlying mechanism of DHI in treating COPD was analyzed using network pharmacology.

**Results:**

A total of 34 eligible studies with a general medium quality were included in the meta-analysis. The pooled data showed that DHI intervention significantly increased clinical efficacy as compared to routine treatment. Meanwhile, our data also revealed that the addition of DHI markedly improved hemorheological indicators, lung function index, arterial blood gas index, and as well as blood coagulation functions. However, the current meta-analysis lacked sufficient data to support the significant effect of DHI on prothrombin time and activated partial thromboplastin time. Network pharmacology found 59 candidate targets of DHI in treating COPD, and enrichment analysis found these targets were associated with lymphocyte proliferation and activation, glucocorticoid receptor signaling, TREM1 signaling, IL-12 signaling and production in macrophages, and aryl hydrocarbon receptor signaling. Multiple core targets including AKT1, TNF, and IL1B, etc. Were identified and might play an important role in the action of DHI against COPD.

**Conclusion:**

Taken together, this study suggested that DHI could ameliorate hemorheological indicators, lung function, arterial blood gas, and as well as coagulation functions of COPD patients and elucidate the underlying mechanism of DHI against COPD.

## 1. Introduction

Chronic obstructive pulmonary disease (COPD), a common respiratory disease characterized by poorly reversible airway obstruction, presents with high global morbidity and mortality.^[1]^ The acute exacerbation of COPD could result in significant adverse consequences for patients, which was associated with increased airway and systemic inflammation and physiological changes. Smoking and air pollution were suggested to be the main risk factors for COPD.^[2,3]^ It was reported that approximately 100 million patients are affected by COPD in China, which causes serious social and economic burden.^[4]^ Currently, medications for COPD included long-acting bronchodilators and a combination of bronchodilators with glucocorticoids.^[5]^ Nevertheless, these medications are always followed by certain adverse effects.^[6]^ Due to the limited acknowledge of the pathogenesis and molecular biology of COPD,^[7]^ the development of novel safe and efficient drugs was time-consuming and laborious.

Traditional Chinese medicine has been developed in China for thousands of years and is effectively used to treat various diseases. It was characterized by multi-components, multi-targets, and multi-pathways in treating diseases. Meanwhile, an excellent safety profile makes it more acceptable. Studies have revealed that various traditional Chinese medicines were effective in ameliorating pulmonary function, inhibiting inflammation, and shortening the acute exacerbation of COPD.^[8–10]^ As an innovative formulation, Chinese herbal injection has been widely employed in the treatment of COPD due to its high bioavailability and rapid action.^[11]^ Danhong injection (DHI) is a commonly used multi-herb Chinese herbal injection with satisfactory efficacy in treating COPD, which was composed of *Salvia miltiorrhiza* Bunge (Danshen in Chinese) and Carthamus tinctorius L. (Honghua in Chinese). Accumulating clinical studies supported the therapeutic effects of DHI in treating COPD or AECOPD, and a meta-analysis was conducted based on 23 randomized controlled trials with 2059 patients to evaluate the influence of DHI in treating COPD.^[12]^ It pooled the clinical efficacy and influences of DHI on several outcome indicators of COPD. However, due to the limitation of the number and quality of included studies, the conclusions still need more high-quality RCT supplementary validation. As increasing high-quality articles are published, an updated meta-analysis was needed. Meanwhile, the elucidation of the underlying mechanism of the action of DHI against COPD was also urgent.

In this study, an updated meta-analysis was performed to evaluate the effects of DHI on improving clinical efficacy and ameliorating hemorheological indicators, lung function, arterial blood gas index, as well as blood coagulation functions of COPD. Further to this, the network pharmacology approach was adopted to investigate the underlying molecular mechanisms of DHI in treating COPD.

## 2. Materials and methods

The preferred reporting items for systematic reviews and meta-analyses criteria were used for this meta-analysis.

### 2.1. Literature search

We performed a computerized literature search of the PubMed, web of science, embase, China national knowledge infrastructure (China national knowledge infrastructure), VIP, and Wanfang databases from their start date to November 8, 2022. We used the following search terms: “chronic obstructive pulmonary disease,” and “Danhong injection,” to obtain relevant articles.

### 2.2. Inclusion and exclusion criteria

Articles were included when meet the following inclusion criteria: randomized controlled trials; using DHI intervention in the experimental group; providing sufficient data for estimating pooled effects with its 95% confidence interval (CI); published in English or Chinese. The exclusion criteria were as follows: patients comorbid with other diseases, reviews, case reports, conference papers, and studies without sufficient data were excluded. If more than 1 article was published using the same subjects, only the study with the largest sample size was selected.

### 2.3. Outcome indicator

Fifteen outcome indicators were evaluated in this study, including clinical efficacy, whole blood viscosity (WBV), low-shear WBV, high-shear WBV, fibrinogen, and hematocrit, forced expiratory volume in 1 second (FEV1), FEV1/Forced vital capacity (FVC), partial pressure of carbon dioxide (PCO2), partial pressure of oxygen (PaO2), arterial oxygen saturation (SaO2), pH, thrombin time (TT), prothrombin time (PT), activated partial thromboplastin time (APTT).

### 2.4. Data extraction and quality assessment

The data were extracted independently by 2 researchers and the dispute was discussed and decided by them. The following data were collected: first author name, publication date, intervention details, sample size, and outcomes indicators. The methodological quality of studies was evaluated according to the scoring standards of the Oxford scoring system (JADAD score). The quality score ranges from 0 to 5 points, with a score of ≤ 2 indicating a low-quality report and a score of ≥ 3 indicating a high-quality report.

### 2.5. Statistical analysis

Statistical analysis was conducted by the Stata SE15.0 software (Stata, China). The risk rate was applied to evaluate the rate of improvement of DHI in the treatment of COPD, and the standard mean difference (SMD) and risk ratio were adopted to assess the pooled effects on other continuous outcome indicators. The heterogeneity across studies was tested using the *I*^2^ statistic, and a random-effect mode was used when *I*^2^ > 50%, otherwise, a fix-effect model was employed. The funnel plot was generated to assess the publication bias. Meta-regression analysis was performed to investigate the source of heterogeneity.

### 2.6. Network pharmacology

The compounds and targets of DHI were retrieved from the (traditional Chinese medicine systems pharmacology database [TCMSP], https://old.tcmsp-e.com/tcmsp.php) database, and those compounds with drug-likeness of ≥ 0.1 were considered as active compounds and used for subsequent analysis. The targets symbol was unified using the UniProt database (https://www.uniprot.org/) and non-human targets were removed. COPD-related genes were identified from the GeneCards (https://www.genecards.org/) and DisGeNET (https://www.disgenet.org/) databases, and the candidate targets of DHI against COPD were obtained by intersecting the COPD-related genes and DHI targets. The protein-protein interaction data of the candidate targets were obtained from the STRING database (https://string-db.org/) and were visualized using the Cytoscape 3.9.1 software. Enrichment analyses were conducted and visualized using the TCGAbiolinks R package.^[13]^ Core bioactive compounds and targets were identified using the cytoHubba plugin in the Cytoscape software.^[14]^

## 3. Results

### 3.1. Search results and basic information of included articles

**Figure 1.**
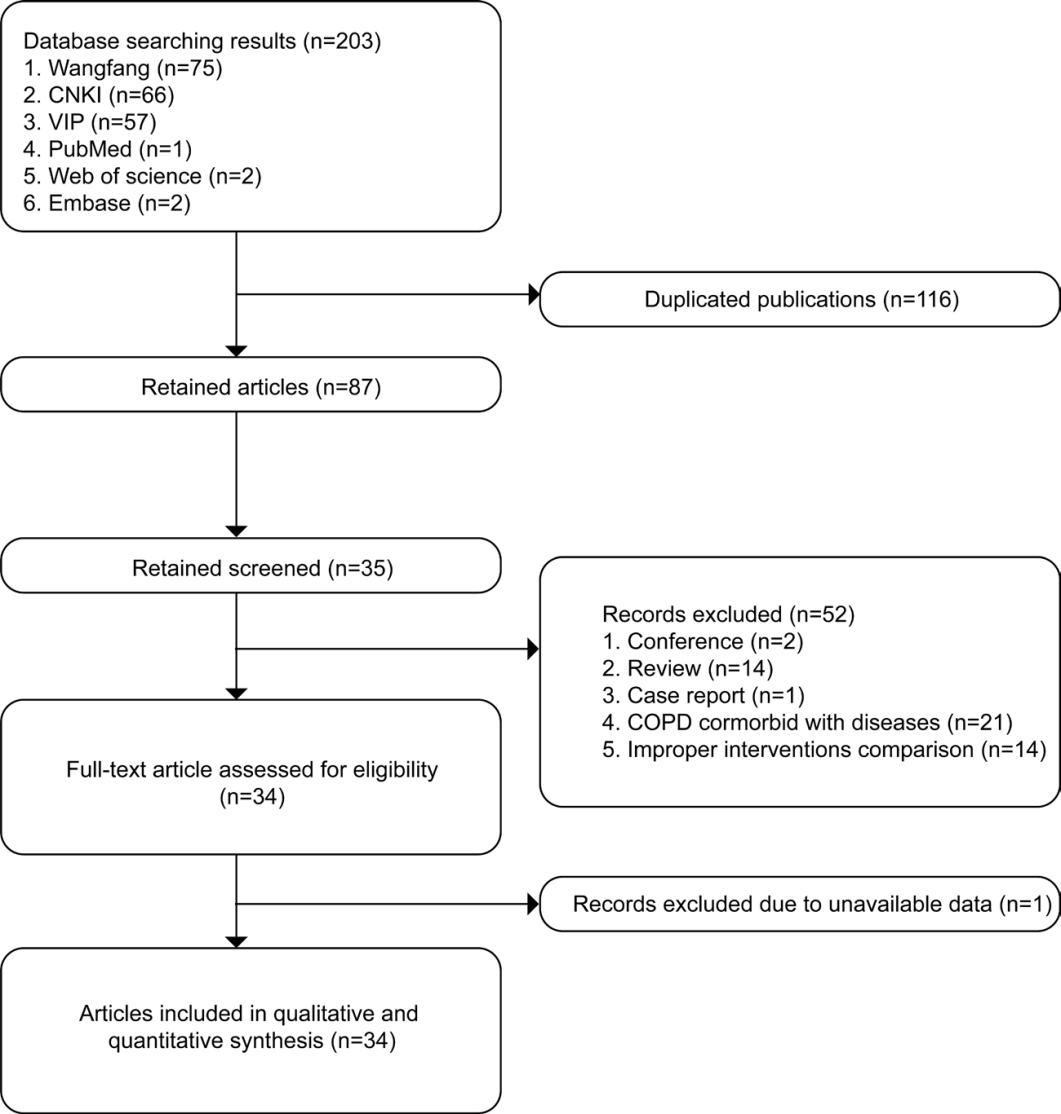
Literature search and screening process.

A total of 203 documents were obtained by initial database searching. Of these, 116 duplications were found and removed, and the rest 87 articles were eliminated by reading the abstracts and titles. Finally, 35 eligible studies were retained for full-text evaluation, which excluded 1 article without available data for meta-analysis. The rest 34 studies were finally included in the meta-analysis.^[15–48]^ Figure 1 showed the literature searching and screening process. The basic information of the 34 studies were shown in Table 1. All studies were conducted in China and published between 2007 and 2022. The sample size ranged from 46 to 306, and the majority of patients were AECOPD. Most studies adopted a similar intervention in the experimental group (DHI, 40 mL/d for 14 days). In terms of outcomes measurement, the clinical efficacy, hemorheological alterations, pulmonary function, arterial blood gas, and coagulation function were generally tested. In addition, 4 studies reported adverse effects and more than half of the studies (29/34) had a high-quality methodology.

**Table 1:** The basic information of included articles.

First Author	Year	Age		case (e.g.,/CG)	Patients	Intervention		Treatment details	Treatment duration	Outcome measures	Adverse effects	Quality
		EG	CG			EG	CG					
Chen Hongci	2007	63–87;78.45	65–85;77.68	40/36	AECOPD	DHI	RT	40 mL	14d	clinical efficacy, high-shear WBV, low-shear WBV, WBV, hematokrit	no	2
Liang Xiao	2009	52.3 ± 2.2	53.1 ± 3.2	52/56	AECOPD	DHI	RT	30mL	14d	hospital day, white blood cell count, PaO2, SaO2, CPIS SCORE	Allergic reactions, gastrointestinal reactions	4
Shi Yiying	2009	52–69;58	51–68;57	23/23	AECOPD	DHI	RT	20–30mL	14d	clinical efficacy, WBV, erythrocyte electrophoresis time, fibrinogen, hematokrit, FEV1, FEV1/FVC	no	3
Tang Tianzhong	2009	43–82;68	40–78;62	68/54	COPD	DHI	RT	40 mL	14d	clinical efficacy, FEV1, FEV1/FVC, high-shear WBV, low-shear WBV, WBV, hematokrit, WBV, hematokrit, fibrinogen, D-dimer, platelet, Dyspnea index	no	4
Liu Zhong	2010	67.7 ± 9.8	66.1 ± 9.2	50/46	AECOPD	DHI	RT	40 mL	14d	clinical efficacy, IL-8, TNF-ɑ	no	2
Yang Ruifang	2010	66.5 ± 5.8	67.2 ± 4.6	42/40	AECOPD	DHI	RT	20 mL	14d	clinical efficacy, blood routine examination, chest X-ray	no	4
Zuo Xiqing	2010	70.35 ± 5.12	70.85 ± 4.04	30/30	AECOPD	DHI	RT	30 mL	14d	clinical efficacy, PaCO2, PaO2, WBV, hematokrit	no	4
Guan Wei	2011	65 ± 4.5	67 ± 3	40/40	AECOPD	DHI	RT	20 mL	14d	low-shear WBV, high-shear WBV, WBV, hematokrit, PT, TT, APTT, fibrinogen, platelet, D-dimer	no	2
Wang Hua	2012	61.2 ± 12.5	65.3 ± 12.7	40/40	AECOPD	DHI	RT	20 mL	14d	clinical efficacy, CRP, MMP-9, TIMP-1, MMP-9/TIMP-1	dizziness	3
Yang Jiewu	2012	N.M	N.M	110/110	AECOPD	DHI	RT	20–30mL	14d	clinical efficacy, FEV1, FEV1/FVC	no	4
Wu Wen	2012	67.8	67.5	30/30	AECOPD	DHI	RT	30 mL	14d	6-min walk test, dyspnea index, FEV1, FEV1/FVC	no	4
Chen Zhuo	2013	66.6 ± 7.4	63.8 ± 7.8	52/48	AECOPD	DHI	RT	40 mL	14d	clinical efficacy, IL-8, TNF-ɑ	no	4
Wang Jinhai	2013	76.35 ± 5.70	69.28 ± 5.18	31/31	AECOPD	DHI	RT	30 mL	14d	clinical efficacy, low-shear WBV, high-shear WBV, WBV, fibrinogen, hematokrit	no	4
Wang Yanli	2013	N.M	N.M	43/42	AECOPD	DHI	RT	40 mL	14d	TCM syndrome score, PaCO2, PaO2, pH, low-shear WBV, high-shear WBV, WBV, hematokrit, SGRQ score	no	2
Xia Jingfen	2013	63 ± 4.2	64 ± 4.5	25/25	AECOPD	DHI	RT	20 mL	15d	low-shear WBV, high-shear WBV, WBV, hematokrit, erythrocyte sedimentation rate, fibrinogen, TT, PT, APTT, D-dimer	no	3
Zhang Li	2013	N.M	N.M	60/60	AECOPD	DHI	RT	20 mL	14d	clinical efficacy	no	3
Du Jin	2014	62 ± 9.8	65 ± 8.4	30/30	AECOPD	DHI	RT	30 mL	14d	clinical efficacy,TT, PT, APTT, fibrinogen, D-dimer	no	3
Qi Chunhui	2014	68.35 ± 5.14	70.25 ± 5.14	65/63	AECOPD	DHI	RT	30 mL	14d	clinical efficacy, low-shear WBV, high-shear WBV, WBV, hematokrit, fibrinogen, PaCO2, PaO2, SaO2	no	3
Yu Shufen	2014	69.5 ± 2.36	68.3 ± 2.14	153/153	COPD	RT ± DHI ± heparin	RT ± heparin	30 mL	10d	clinical efficacy, FEV1, FEV1/FVC, PaCO2, PaO2	no	4
Wang Jialie	2014	63.3 ± 4.4	61.4 ± 5.3	50/50	COPD	RT ± DHI	RT	40 mL	14d	clinical efficacy, pH, PaCO2, PaO2, WBV, hematokrit, erythrocyte sedimentation rate, fibrinogen,	no	4
Zhang Qiong	2014	64.45 ± 9.46	64.41 ± 9.45	64/64	AECOPD	RT ± DHI	RT	40 mL	14d	clinical efficacy, low-shear WBV, high-shear WBV, WBV	no	
Zhu Renqian	2014	62.5 ± 7.92	61.5 ± 8.84	40/40	AECOPD	RT ± ambroxol ± DHI	RT ± ambroxol	40 mL	15d	clinical efficacy, FEV1, FEV1/FVC	no	4
Ge Zongkai	2016	54.9 ± 4.4	55.7 ± 4.6	32/32	COPD	RT ± DHI	RT	40 mL	14d	clinical efficacy, WBV, erythrocyte electrophoresis time, fibrinogen, hematokrit	no	4
Zhao Liang	2016	59.6 ± 7.9	58.1 ± 8.2	50/50	AECOPD	RT ± DHI	RT	30 mL	14d	Thrnmbomodulin, vWF, D-dimer, PT, APTT, TT, fibrinogen	no	3
Jia Zhongrui	2017	62.7 ± 6.1	63.5 ± 5.8	62/62	AECOPD	RT ± DHI	RT	20 mL	10d	clinical efficacy, D-dimer, APTT, TT, fibrinogen	no	4
Shen Qing	2017	65.4 ± 6.4	65.8 ± 7.3	56/56	AECOPD	RT ± DHI	RT	40 mL	14d	clinical efficacy, pH, PaCO2, PaO2, IgG, IgK, IgM, INF-γ, TNF-ɑ, IL-10, T lymphocyte subsets	Palpitations, nausea, headaches	4
Yang Aiping	2018	N.M	N.M	98/98	AECOPD	RT ± DHI ± phentolamine	RT ± phentolamine	20 mL/d	7d	clinical efficacy, Time of symptom resolution	no	2
Yang Yuxia	2018	66.2 ± 8.0	66.9 ± 7.6	118/118	AECOPD	RT ± DHI	RT	2 × 40 mL	4d	clinical efficacy, TCM syndrome score, PaCO2, PaO2, SaO2, sE-selection, vWF, APTT, D-dimer, platelet, thromboelastography	no	4
Liu Xinyan	2019	64.0 ± 4.68	62.5 ± 3.72	30/30	AECOPD	DHI	RT	40 mL	14d	lipid peroxidase, glutathione peroxidase, catalase, TNF- α, IL-6, CRP, FEV1, FEV1/FVC	no	5
Wang Jiazhen	2019	52.3 ± 7.6	52.1 ± 7.5	60/60	AECOPD	RT ± DHI	RT	20 mL	14d	clinical efficacy, S100A12, procalcitonin, CRP, IL-1β, IL-8, TNF-ɑ, pH, PaCO2, PaO2, SaO2, FEV1, FEV1/FVC, left ventricular ejection fraction, cardiac output, pulmonary artery systolic pressure, right ventricular outflow tract	no	5
Chen Xiuhong	2020	58.11 ± 4.02	59.03 ± 3.98	37/37	COPD	RT ± DHI ± Montelukast	RT ± Montelukast	40 mL	14d	clinical efficacy, FEV1, FEV1/FVC, procalcitonin, hematokrit, fibrinogen	Vomiting, nausea, dizziness	4
Huang Yajing	2021	68.83 ± 4.41	68.32 ± 4.38	33/32	AECOPD	RT ± DHI	RT	2 × 40 mL	30d	clinical efficacy, PaCO2, PaO2, SaO2, APTT, D-dimer, platelet, sE-selevtion, vWF	no	4
Zheng Shenghua	2022	79.5 ± 8.5	79.4 ± 8.3	40/40	AECOPD	RT ± DHI	RT	40 mL	7d	clinical efficacy, PaCO2, PaO2, FEV1, FEV1/FVC, white blood cell count, neutral cell count, CRP, AT, D-dimer, fibrinogen, hemoglobin, red blood cells, platelet, alanine aminotransferase, serum creatinine, blood urea nitrogen	no	4
Gao Xiaoyu	2022	71.7 ± 8.2	72.2 ± 6.7	56/56	AECOPD	RT ± DHI	RT	40 mL	10d	clinical efficacy, PaCO2, PaO2, SaO2, FEV1, FEV1/FVC, TCM syndrome score	no	5

AECOPD = acute exacerbation of chronic obstructive pulmonary disease, APTT = activated partial thromboplastin time, CG = control group, COPD = chronic obstructive pulmonary disease, CRP = C-reactive protein, DHD = Danhong injection, EG = experimental group, FCV = forced vital capacity, FEV1 = forced expiratory volume in 1 second, IL-10 = interleukin 10, IL-8 = interleukin 8, MMP-9 = matrix metallopeptidase 9, PaCO2 = partial pressure of carbon dioxide, PaO2 = partial pressure of oxygen, PT = prothrombin time, RT = routine treatment, SaO2 = arterial oxygen saturation, TCM = traditional Chinese medicine, TIMP-1 = TIMP metallopeptidase inhibitor 1, TNF-α = tumor necrosis factor alpha, TT = thrombin time, vWF = von willebrandfactor, WBV = whole blood viscosity.

### 3.2. Comparison of clinical efficacy

**Figure 2.**
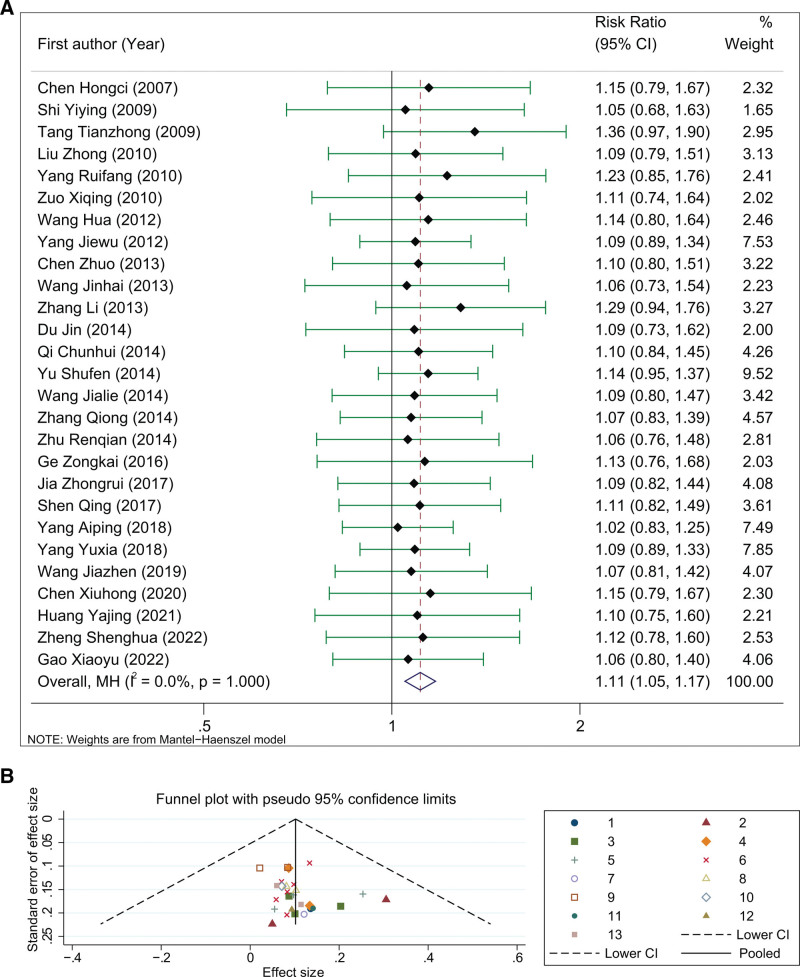
The meta-analytic results of the clinical efficacy of DHI in treating COPD. COPD = chronic obstructive pulmonary disease, DHI = Danhong injection.

The clinical efficacy was reported in 27 studies, with 1540 and 1509 cases in the experimental and control groups, respectively. The pooled results were calculated using a fix-effect model due to the insignificant heterogeneity across included studies (*I*^2^ = 0.0%, *P* = 1.000), and demonstrated in almost all of these studies, the pooled risk ratio was 1.11 (95% CI: 1.05–1.17), and the difference was statistically significant (Z = 3.627 and *P* < .0001, Fig. 2A). It was observed that the funnel plot of the included studies was symmetrical with the midline, suggesting that the research accuracy was high and that there was no publication bias (Fig. 2B).

### 3.3. Comparison of hemorheological indicators

**Figure 3.**
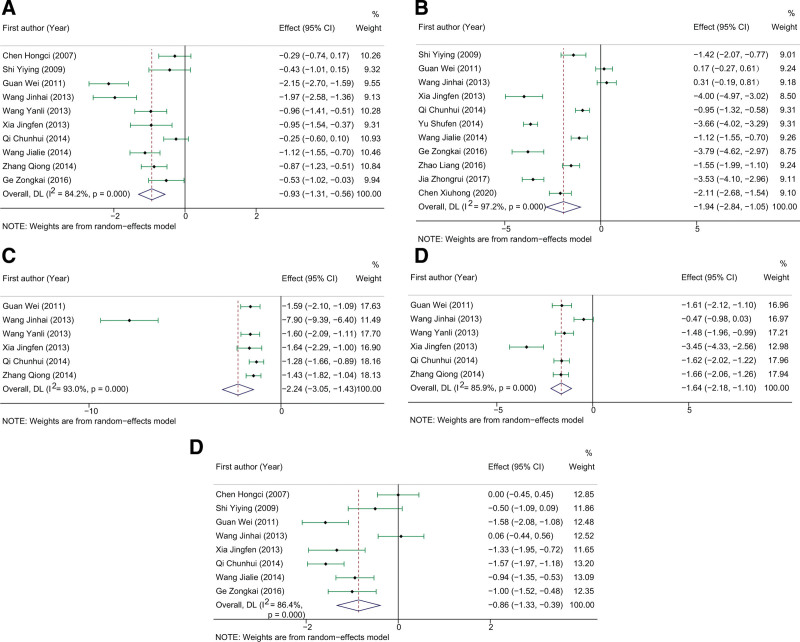
The pooled results of the influence of DHI on hemorheological indicators of COPD patients. (A-D) represented the pooled SMD of WBV, fibrinogen, high-shear WBV, low-shear WBV, and hematocrit, respectively. COPD = chronic obstructive pulmonary disease, DHI = Danhong injection, SMD = standard mean difference, WBV = whole blood viscosity.

To compare the differences in hemorheological indicators between the experimental and control groups, we evaluated the pooled effects of WBV, low-shear WBV, high-shear WBV, fibrinogen, and hematocrit. As shown in Figure 3A, ten studies containing 819 patients reported the WBV data and were used to conduct a meta-analysis. The overall heterogeneity was significant (*I*^2^ = 84.2%, *P* < .0001), and the random effect model was applied to analyze the whole. The pooled results demonstrated that the WBV in the experimental group was significantly lower than that in the control group (SMD: −0.93, 95%CI: −1.31 to −0.56, Z = −11.307, *P* < .0001). In addition, the pooled data also revealed a significantly lower fibrinogen (SMD: −1.94, 95%CI: −2.84 to −1.05, Z = −4.257, *P* < .0001, Fig. 3B), high-shear WBV (SMD: −2.24, 95%CI: −3.05 to −1.53, Z = −5.416, *P* < .0001, Fig. 3C), shear-WBV (SMD: −1.64, 95%CI: −2.18 to −1.10, Z = −5.968, *P* < .0001, Fig. 3D), and hematocrit (SMD: −0.86, 95%CI: −1.33 to −0.39, Z = −3.595, *P* < .0001, Fig. 3E) in the experimental group as compared to that in the control group. All pooled effects were calculated using a random effect model due to the heterogeneity across studies.

### 3.4. Comparison of lung function indexes

**Figure 4.**
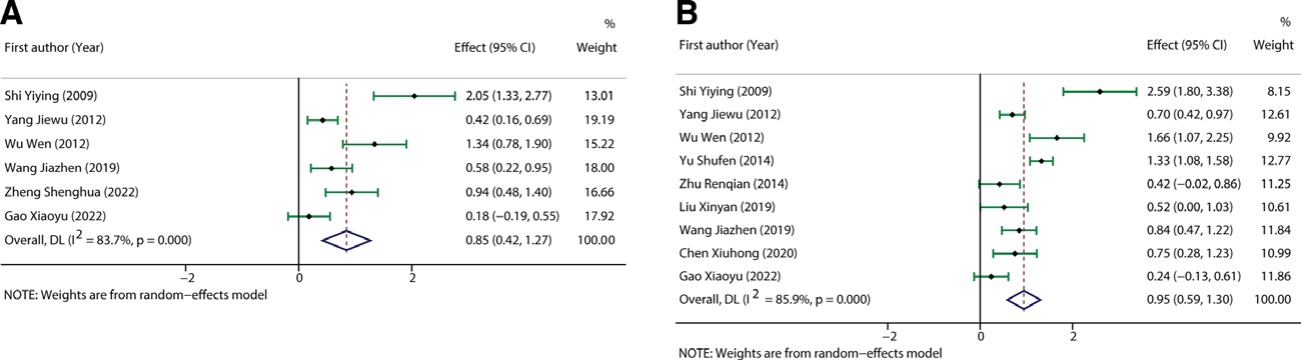
The pooled results of the influence of DHI on lung function index of COPD patients. (A and B) represented the pooled SMD of FEV1 and FEV1/FVC, respectively. COPD = chronic obstructive pulmonary disease, DHI = Danhong injection, FVC = Forced vital capacity, SMD = standard mean difference.

The pulmonary function was assessed by calculating the pooled effects of FEV1 and FEV1/FVC between the experimental and control groups. In terms of FEV1, 6 studies reported relevant data and were included in the meta-analysis. The random-effect model was adopted due to the higher heterogeneity across studies (*I*^2^ = 83.7%, *P* < .0001), and the pooled data demonstrated that DHI intervention significantly elevated the FEV1 as compared to routine treatment. (SMD: 0.85, 95%CI: 0.42–1.27, Z = 3.909, *P* < .0001, Fig. 4A). In addition, 9 studies reported FEV1/FVC data of 1078 patients with COPD, and significant heterogeneity was observed among studies (*I*^2^ = 85.9%, *P* < .0001). The pooled results revealed that the FEV1/FVC in the experimental group was markedly increased as compared to the control group (SMD: 0.95, 95%CI: 0.59–1.30, Z = 5.198, *P* < .0001, Fig. 4B).

### 3.5. Comparison of arterial blood gas indexes

**Figure 5.**
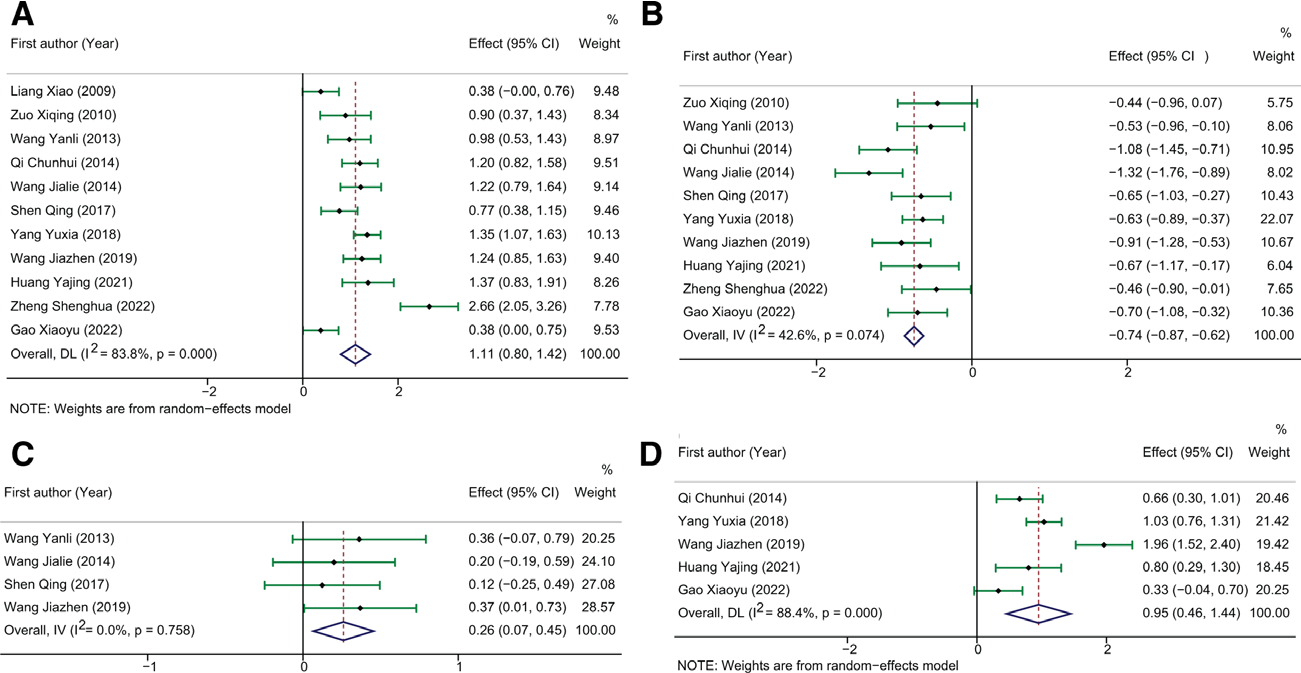
The pooled results of the influence of DHI on arterial blood gas index of COPD patients. (A-D) represented the pooled SMD of PaO2, PCO2, pH, and SaO2, respectively. COPD = chronic obstructive pulmonary disease, DHI = Danhong injection, PCO2 = partial pressure of carbon dioxide, SaO2 = arterial oxygen saturation, SMD = standard mean difference.

Four arterial blood gas indexes including PCO2, PaO2, SaO2, and pH were included for meta-analysis. As shown in Figure 5A, eleven studies provided available data on PaO2 and presented with high heterogeneity (*I*^2^ = 83.8%, *P* < .0001). The meta-analytic results showed that administration of DHI for patients with COPD significantly increased PaO2 as compared to the routine treatment (SMD: 1.11, 95%CI: 0.80–1.42, Z = 6.998, *P* < .0001). The pooled effects on PCO2 and pH were calculated under fix-effect models, and the results showed a significantly decreased PCO2 (SMD: −0.74, 95%CI: −0.87 to −0.62, Z = −11.875, *P* < .0001, Fig. 5B) and increased pH (SMD: 0.26, 95%CI: 0.07–0.45, Z = 2.648, *P* = .008, Fig. 5C) in the experimental group as compared to the control group. A random effect model was adopted to calculate the pooled effects of SaO2, and the results showed that DHI treatment significantly elevated the SaO2 of COPD patients as compared to the routine treatment (SMD: 0.95, 95%CI: 0.46–1.44, Z = 11.150, *P* = .008, Fig. 5D).

### 3.6. Comparison of blood coagulation indexes

**Figure 6.**
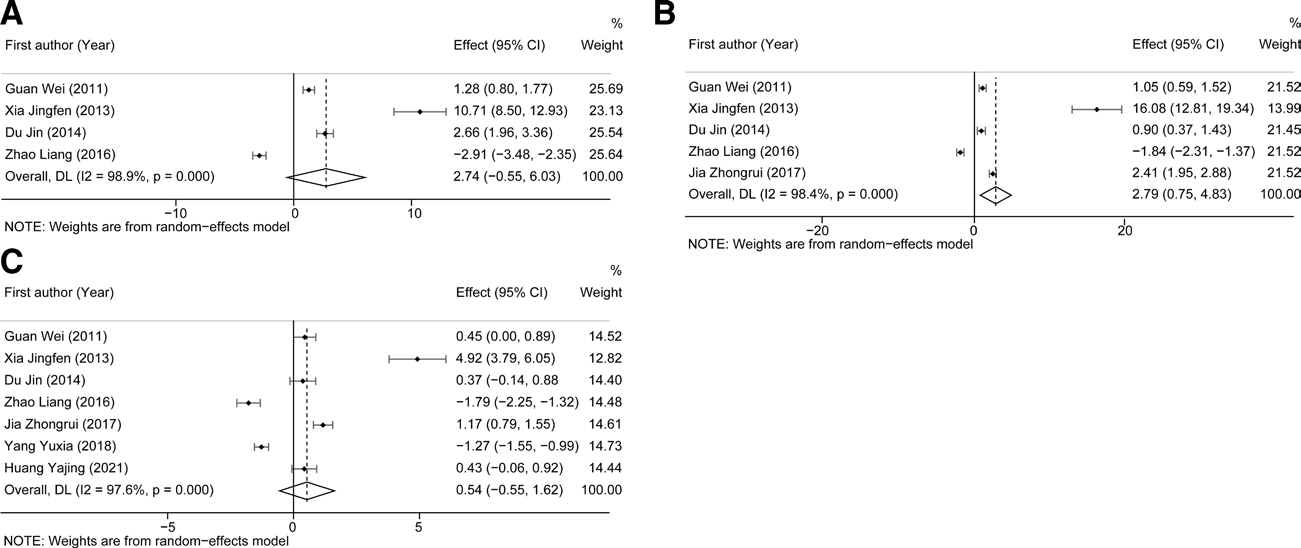
The pooled results of the influence of DHI on blood coagulation indexes of COPD patients. (A-C) represented the pooled SMD of PT, TT, and APTT, respectively. APTT = activated partial thromboplastin time, COPD = chronic obstructive pulmonary disease, DHI = Danhong injection, PT = prothrombin time, SMD = standard mean difference, TT = thrombin time.

Three indexes including PT, TT, and APTT were used to evaluate pooled effects of DHI on blood coagulation functions. As shown in Figure 6A, 4 studies with 290 COPD patients reported available data on PT, and high heterogeneity was observed across studies (*I*^2^ = 98.9%, *P* < .0001). The pooled results revealed an insignificantly higher level of PT in the experimental group as compared to the control group (SMD: 2.74, 95%CI: −0.55 to 6.03, Z = 1.630, *P* = .103). In terms of TT, the pooled results showed a significantly higher TT level in the experimental group as compared to the control group (SMD: 2.79, 95%CI: 0.75–4.83, Z = 2.680, *P* = .007, Fig. 6B). In addition, insignificant pooled effects on APTT were also observed between the 2 groups (SMD: 0.54, 95%CI: −0.55 to 1.62, Z = 0.964, *P* = .335, Fig. 6C).

### 3.7. Meta-regression analysis

Heterogeneity was observed across studies when evaluating the pooled influences of Danhong injection on outcomes of COPD. Therefore, meta-regression analysis was performed to explore the source of heterogeneity. As shown in Table 2, we assessed the contribution of the number of cases, dosage, duration, and combination of dosage and duration to the heterogeneity, and it was found that the number of cases was a contributor to the heterogeneity of FEV1 and the heterogeneity when analyzing fibrinogen was attributed to duration. However, these factors did not contribute to heterogeneity in the analyses of other outcomes.

**Table 2:** The results of meta-regression analysis on evaluated variables

Variables	SMD	Coefficient	Standard error	*t*	*P*
Low-shear WBV	case	0.4102257	0.5316285	0.77	.521
	dosage	−0.2567426	0.5339007	−0.48	.678
	duration	2.346904	0.7959273	2.95	.098
WBV	case	−0.2400809	0.5237196	−0.46	.663
	dosage	0.3866834	0.5032922	0.77	.471
	duration	−0.2893227	0.8594685	−0.34	.748
High-shear WBV	case	−2.006294	3.102205	−0.65	.584
	dosage	1.683941	3.102764	0.54	.642
	duration	−2.557254	4.016248	−0.64	.589
Fibrinogen	case	−0.1071032	0.6183996	−0.17	.867
	dosage	−1.593431	0.7175171	−2.22	.062
	duration	3.063377	0.7430993	4.12	**.004**
Hematocrit	case	0.6872588	0.5803166	1.18	.302
	dosage	0.3209759	0.5349864	0.6	.581
	duration	0.6028882	0.819984	0.74	.503
FEV1	case	0.9778324	0.2135377	4.58	**.02**
	dosage	−0.4118172	0.200968	−2.05	.133
FEV1/FVC	case	0.8730151	0.3632285	2.4	.074
	dosage	−1.417356	0.5118696	−2.77	.05
	duration	−0.406218	0.4605663	−0.88	.428
	dosage_duration	0.2632677	0.6195555	0.42	.693
PT		insufficient observations			
TT		insufficient observations			
APTT	case	2.456392	1.388916	1.77	.152
	dosage	−1.992919	1.389979	−1.43	.225
PaO2	case	0.4503308	0.428293	1.05	.334
	dosage	0.0812959	0.6643012	0.12	.907
	duration	−0.3257211	0.5781588	−0.56	.594
	dosage_duration	−0.0669454	0.8336396	−0.08	.939
PCO2	case	0.3096247	0.178157	1.74	.143
	dosage	0.0150301	0.2554227	0.06	.955
	duration	−0.1894276	0.2308398	−0.82	.449
	dosage_duration	−0.0040817	0.3346344	−0.01	.991
pH		insufficient observations			
SaO2		insufficient observations			

APTT = activated partial thromboplastin time, FEV1 = forced expiratory volume in 1 second, FVC = FEV1/Forced vital capacity, PaO2 = partial pressure of oxygen, PCO2 = partial pressure of carbon dioxide, PT = prothrombin time, SaO2 = arterial oxygen saturation, SMD = standard mean difference, TT = pH, thrombin time, WBV = whole blood viscosity.

### 3.8. Active compounds and targets of DHI

**Figure 7.**
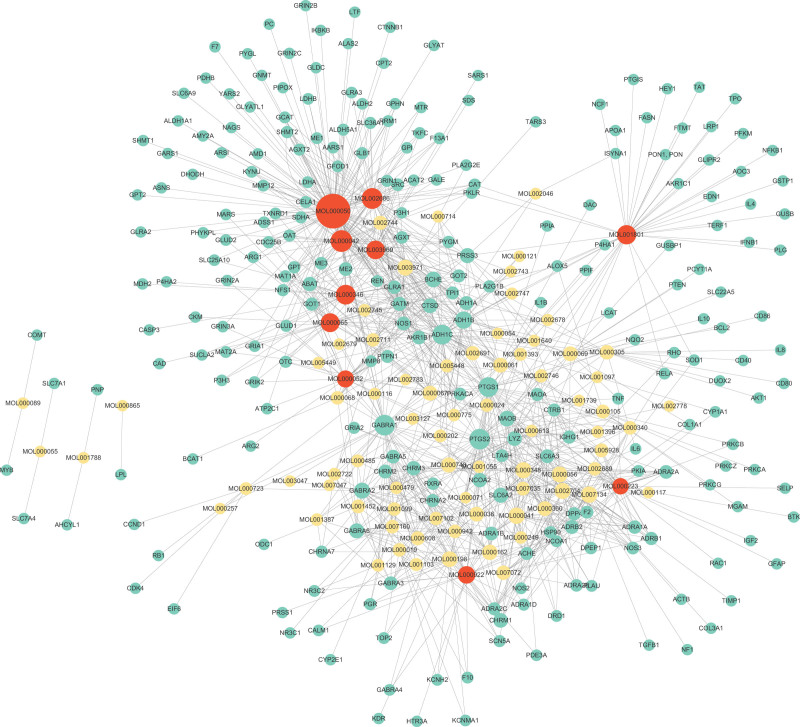
The network contains the connections among active compounds and their targets.

To elucidate the underlying mechanism of DHI against COPD, we first retrieved the chemical constituents and targets of DHI. As a result, we found 376 chemical constituents of DHI from the TCMSP database, including 202 from Danshen and 189 from Honghua. Further, 124 compounds were identified as bioactive compounds due to their drug-likeness ≥ 0.1 (Table S1, Supplemental Digital Content, http://links.lww.com/MD/I423). Therefore, we collected the targets of these bioactive compounds from the TCMSP database and removed those that were not human genes. As shown in Figures 7, 85 bioactive compounds of DHI targeted 249 proteins belonging to human (Table S2, Supplemental Digital Content, http://links.lww.com/MD/I424). Table 3 showed the top 10 core bioactive compounds arranged by the degree value, which had the most number of targets and might be crucial for the action of DHI against COPD.

**Table 3:** The main topological characteristics of the top ten core bioactive compounds arranged by degree value.

MOL_ID	Compounds	Betweenness Centrality	Closeness Centrality	Degree	Neighborhood Connectivity
MOL000050	2-Azaniumylacetate	0.32	0.47	105	5.75
MOL000042	(2S)-2-azaniumylpropanoate	0.05	0.40	47	8.55
MOL002686	Glyoxylic acid	0.03	0.38	43	8.14
MOL000346	Succinic Acid	0.05	0.38	38	8.87
MOL001801	Salicylic acid	0.15	0.38	36	7.08
MOL000065	(3S)-3-azaniumyl-4-hydroxy-4-oxobutanoate	0.04	0.38	34	9.03
MOL003969	d,l-Serine	0.04	0.39	33	10.91
MOL000922	(-)-Terpinen-4-ol	0.04	0.38	28	11.96
MOL000052	(2S)-2-azaniumyl-5-hydroxy-5-oxopentanoate	0.03	0.38	26	11.81
MOL007134	Danshensu	0.04	0.34	23	8.96

### 3.9. Candidate targets of DHI against COPD

**Figure 8.**
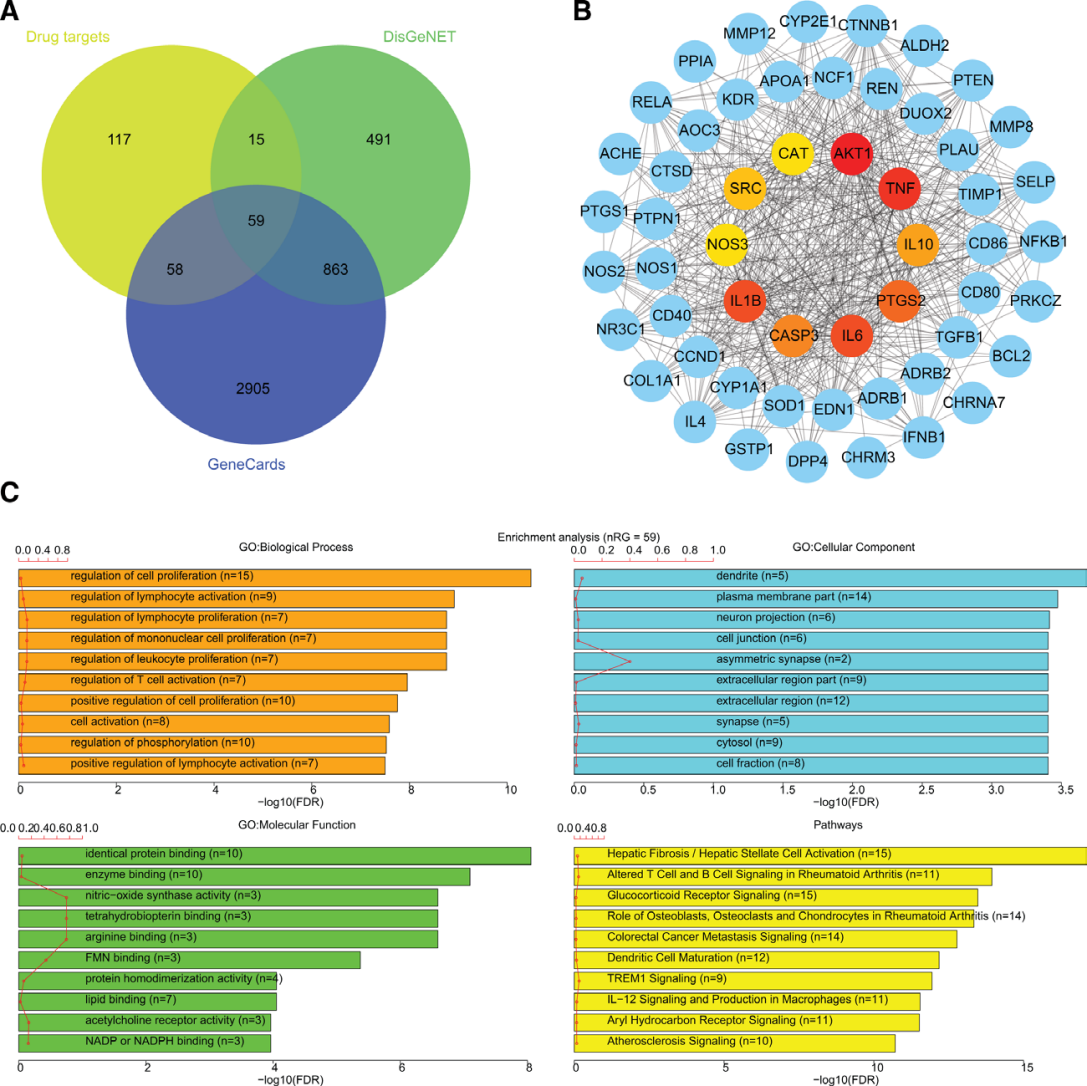
The candidate targets of DHI against COPD. (A) The Venn diagram of the COPD-related genes and DHI targets; (B) The PPI network of the candidate targets; (C) The top ten enriched terms of biological process, cellular components, molecular function, and pathways with a lower FDR value. DHI = Danhong injection, COPD = chronic obstructive pulmonary disease, FDR = false discovery rat, PPI = protein-protein interaction.

After data mining, 922 genes were obtained to be associated with COPD, and 59 candidate targets were identified by overlapping these genes with DHI targets (Fig. 8A). Figure 8B illustrated the protein-protein interaction network of the candidate targets, which contains 57 nodes and 531 edges. The core targets were identified by the cytoHubba plugin and the top 10 targets were illustrated in Table 4. GO and KEGG enrichment analyses demonstrated that these candidate targets were significantly enriched in 315 biological processes, 41 cellular components, 53 molecular functions, and 190 pathways (Table S3, Supplemental Digital Content, http://links.lww.com/MD/I425). Figure 8C showed the top 10 terms with lower false discovery rate values. It suggested that the candidate targets were mainly involved in lymphocyte proliferation and activation. Meanwhile, multiple pathways including glucocorticoid receptor signaling, TREM1 signaling, IL-12 signaling and production in macrophages, and aryl hydrocarbon receptor signaling were also associated with these candidate targets.

**Table 4:** The main topological characteristics of the top 10 core targets arranged by degree value.

Symbol	Gene name	Betweenness Centrality	Closeness Centrality	Degree	Neighborhood Connectivity
AKT1	AKT serine/threonine kinase 1	0.13	0.91	48	19.56
TNF	tumor necrosis factor	0.08	0.90	47	20.23
IL1B	Interleukin-1 beta	0.07	0.88	46	20.48
IL6	Interleukin-6	0.10	0.88	46	20.37
PTGS2	Prostaglandin-endoperoxide synthase 2	0.04	0.80	40	22.18
CASP3	Caspase-3	0.04	0.77	37	22.78
IL10	Interleukin-10	0.02	0.75	35	23.89
SRC	SRC proto-oncogene, non-receptor tyrosine kinase	0.02	0.73	33	24.70
CAT	Catalase	0.03	0.70	30	23.37
NOS3	Nitric oxide synthase, endothelial	0.02	0.70	30	24.57

## 4. Discussion

Since a meta-analysis was conducted in 2019, accumulating studies provided additional evidence to evaluate the clinical efficacy of DHI in treating COPD, as well as the influences of DHI on other outcome indicators of COPD. Therefore, this study pooled the data of recently published data and the last meta-analysis and comprehensively analyzed the impacts of DHI on hemorheological alterations, pulmonary function, arterial blood gas, and coagulation function of patients with COPD. Subsequently, network pharmacology was employed to elucidate the pharmacological mechanism of DHI in the treatment of COPD to make up for its theoretical deficiencies for clinical application.

Consistent with the previous meta-analysis, our data revealed a significantly improved clinical efficacy of DHI in treating COPD as compared to the control group. A basically symmetrical funnel plot indicated no publication bias, and the heterogeneity of the papers was not observed. COPD progression could result in significant adverse consequences such as systemic inflammation and physiological changes. Herein, the 34 studies included in the meta-analysis reported the influence of DHI on a variety of indexes, including hemorheological alterations, pulmonary function, arterial blood gas, and coagulation function. In the present study, we selected and assessed fifteen outcome indicators including clinical efficacy and the above domains. Besides, part of these studies provided available data on inflammation, platelet, and immune cells.

Chronic obstructive pulmonary disease is characterized by the decreased maximal volume of gas and increased residual volume. With the development of the disease, the vascular bed area after lung injury decreases and the pulmonary vascular constriction forms a state of hypoxia and ischemia in the body. This study evaluated the improvement of 2 indicators (FEV1 and FEV1/FVC) after DHI as compared to the control group. The results revealed a favorable improvement by DHI as compared to routine treatment. Therefore, DHI was beneficial for improving lung function. Cryptotanshinone, a lipophilic compound extracted from the root of Danshen, could protect against pulmonary fibrosis by inhibiting Smad and STAT3 signaling pathways.^[49]^ In addition, tanshinones from Danshen also exert pharmacological activities in ameliorating pulmonary diseases.^[50]^ It was reported that hydroxysafflor yellow A, a main constituent of Honghua, showed vasodilatation effects on the pulmonary artery^[51]^ and can attenuate lung injury by inhibiting platelet activation.^[52]^ COPD is characterized by airflow limitation which is progressive in the course of illness, and by the changes in arterial blood gases that can lead to respiratory failure.^[53]^ The pooled data showed DHI administration could make that course slower by decreasing PCO2 and increasing PaO2 and pH, and SaO2. Hydroxysafflor yellow A from the flower of Carthamus tinctorius L. was revealed to ameliorate the alterations in arterial blood gases in mice with acute lung injury.^[54]^

Modern medical research has found that COPD and hypoxemia can increase the aggregation of red blood cells, damage vascular endothelial cells, activate blood clotting factors, lead to the increase of clotting substances, lead to the increase of whole blood viscosity, the formation of a hypercoagulable state. Herein, it was observed that DHI was beneficial for ameliorating hemorheological alterations and coagulation function in COPD patients. The main manifestations in the DHI-treated groups were decreased blood viscosity, fibrinogen and hematocrit and increased thrombin time. Blood viscosity is a significant factor that plays an important role in pulmonary and cardiovascular diseases.^[55]^ The water-soluble extracts of Danshen have been reported to improve abnormal hemorheological parameters including blood viscosity and viscoelasticity in aging guinea pigs.^[56]^ Fibrinogen is an FDA-qualified prognostic biomarker in COPD, and elevated fibrinogen was associated with a higher risk of mortality.^[57]^ The inhibition of fibrinogen by DHI might be partially attributed to phenolic acids obtained from Danshen.^[58]^ Hematocrit is negatively associated with mortality and morbidity of COPD,^[59]^ and the carthamins yellow from Honghua was responsible for this induction of hematocrit by DHI.^[60]^ The available data do not provide sufficient evidence to determine the effect of DHI on PT and APTT in COPD patients. This phenomenon may be caused by the significant heterogeneity among samples and the conflicting effects of DHI components on coagulation function.^[58,61,62]^

Given the influence of DHI on various outcome indicators of COPD, it is urgent to elucidate the underlying mechanism. Herein, a total of 59 candidate targets were identified to contribute to the anti-COPD action of DHI, and the protein-protein interaction topological characteristics showed that AKT1, TNF and IL6 might be pivotal in the treatment of COPD by DHI. AKT1 is a serine-threonine protein kinase that is involved in various crucial signal transduction and biological processes. Modern pharmacological research demonstrated that AKT1 was regulated by various compounds from DHI, such as tanshinone.^[63]^ Hydroxysafflor yellow A has been reported to alleviate increased TNF and IL6, leading to the inhibition of inflammation in patients with COPD.^[64]^ In addition, it was observed the action of DHI against COPD was associated with the regulation of multiple pathways, such as glucocorticoid receptor signaling, TREM1 signaling, and production in macrophages. COPD is associated with loss of GCR in senescent CD28null and NKT-like cells^[65]^ and synthetic glucocorticoids are widely prescribed drugs for COPD.^[66]^ A recent study demonstrated that TREM-1 aggravates the development of COPD via activating NLRP3 inflammasome-mediated pyroptosis.^[67]^ Macrophages circulate in the blood and control innate and acquired immunity, as well as homeostasis. It plays a crucial role in the pathogenesis of COPD by different polarization.^[68]^

## 5. Conclusion

In the present study, we pooled the data of various outcome indicators in COPD patients treated by DHI. The results demonstrated that DHI administration significantly improved the clinical efficacy and ameliorated alterations of hemorheology and arterial blood gas, and alleviate coagulation and pulmonary functions. Network pharmacology analysis revealed the potential targets and pathways of the action of DHI against COPD. However, further experimental studies are required to validate these findings.

## Acknowledgments

We are grateful to all researchers in the enrolled studies.

## Author contributions

**Conceptualization:** Xiaoyu Gao, Jinsong Gao.

**Data curation:** Xiaoyu Gao, Jinsong Gao.

**Formal analysis:** Xiaoyu Gao, Jinsong Gao.

**Methodology:** Xiaoyu Gao, Jinsong Gao.

**Software:** Xiaoyu Gao, Jinsong Gao.

**Visualization:** Xiaoyu Gao, Jinsong Gao.

**Writing – original draft:** Xiaoyu Gao, Jinsong Gao.

**Writing – review & editing:** Xiaoyu Gao, Jinsong Gao.

## Supplementary Material






